# Identification and validation of a novel HOX-related classifier signature for predicting prognosis and immune microenvironment in pediatric gliomas

**DOI:** 10.3389/fcell.2023.1203650

**Published:** 2023-07-21

**Authors:** Jiao Zhang, Xueguang Zhang, Junyan Su, Jiali Zhang, Siyao Liu, Li Han, Mengyuan Liu, Dawei Sun

**Affiliations:** ^1^ Department of Cardiology, Capital Medical University Electric Power Teaching Hospital, State Grid Beijing Electric Power Hospital, Beijing, China; ^2^ Department of Nephrology, Capital Medical University Electric Power Teaching Hospital, State Grid Beijing Electric Power Hospital, Beijing, China; ^3^ Beijing ChosenMed Clinical Laboratory Co Ltd., Beijing, China

**Keywords:** HOX family genes, prognosis, immune infiltrates, signature, pediatric gliomas

## Abstract

**Background:** Pediatric gliomas (PGs) are highly aggressive and predominantly occur in young children. In pediatric gliomas, abnormal expression of Homeobox (HOX) family genes (HFGs) has been observed and is associated with the development and progression of the disease. Studies have found that overexpression or underexpression of certain HOX genes is linked to the occurrence and prognosis of gliomas. This aberrant expression may contribute to the dysregulation of important pathological processes such as cell proliferation, differentiation, and metastasis. This study aimed to propose a novel HOX-related signature to predict patients’ prognosis and immune infiltrate characteristics in PGs.

**Methods:** The data of PGs obtained from publicly available databases were utilized to reveal the relationship among abnormal expression of HOX family genes (HFGs), prognosis, tumor immune infiltration, clinical features, and genomic features in PGs. The HFGs were utilized to identify heterogeneous subtypes using consensus clustering. Then random forest-supervised classification algorithm and nearest shrunken centroid algorithm were performed to develop a prognostic signature in the training set. Finally, the signature was validated in an internal testing set and an external independent cohort.

**Results:** Firstly, we identified HFGs significantly differentially expressed in PGs compared to normal tissues. The individuals with PGs were then divided into two heterogeneous subtypes (HOX-SI and HOX-SII) based on HFGs expression profiles. HOX-SII showed higher total mutation counts, lower immune infiltration, and worse prognosis than HOX-SI. Then, we constructed a HOX-related gene signature (including *HOXA6*, *HOXC4*, *HOXC5*, *HOXC6*, and *HOXA-AS3*) based on the cluster for subtype prediction utilizing random forest supervised classification and nearest shrunken centroid algorithm. The signature was revealed to be an independent prognostic factor for patients with PGs by multivariable Cox regression analysis.

**Conclusion:** Our study provides a novel method for the prognosis classification of PGs. The findings also suggest that the HOX-related signature is a new biomarker for the diagnosis and prognosis of patients with PGs, allowing for more accurate survival prediction.

## 1 Introduction

Gliomas are the most common central nervous system (CNS) tumors in children, accounting for the vast majority of malignant brain tumors. Pediatric gliomas (PGs) are clinically and biologically distinct from adult gliomas (AGs) (Ryall et al., 2017). It is crucial to gain a better understanding of the genetic and molecular abnormalities underlying the disease for early diagnosis, appropriate treatment, and improved prognosis in PGs patients. Most pediatric gliomas present as benign, slow-growing lesions classified as grade I or II by the WHO classification of CNS tumors (Louis et al., 2021). These low-grade gliomas (LGGs) account for approximately 30% of pediatric CNS tumors (Ostrom et al., 2018). In contrast to adult LGGs, IDH mutations are almost absent in children, and malignant progression in pediatric LGGs is sporadic and has excellent overall survival (OS) under current treatment strategies (Sturm et al., 2017). Surgical excision is the mainstay of current therapy for LGG, which may be curative where total resection is possible (Diwanji et al., 2017). However, there is still a risk of progression or relapse. Moreover, a significant proportion of gliomas exhibit rapid growth and progression, thus classified as WHO grade III or IV high-grade gliomas (HGGs) (Sturm et al., 2017). Pediatric HGGs account for 8%–12% of pediatric CNS tumors and may manifest across all ages and anatomic CNS compartments (Funakoshi et al., 2021). Somatic mutations in histone genes, specifically K27M and G34R/V mutations in H3.3- and H3.1- coding genes, have been identified as hallmarks of HGGs in children and young adults. BRAF V600E mutations are found in 5%–10% of pediatric HGGs (Funakoshi et al., 2021).

The investigation of gene families in tumors is a prominent and dynamic field of research in cancer studies. Gene families play a crucial role in various biological processes, including tumorigenesis and tumor progression. Understanding the involvement of gene families in cancer provides valuable insights into the molecular mechanisms underlying tumor development and opens new avenues for therapeutic interventions. Gene families consist of a group of genes that share similar sequences or functions. In the context of cancer, alterations within gene families can have significant implications for tumor initiation, growth, and response to treatment. The study of gene families in tumors focuses on identifying specific gene family members that are dysregulated or mutated in cancer cells, as well as investigating their functional roles and interactions within cellular pathways. By unraveling the role of gene families in cancer, researchers can identify potential biomarkers for early detection, prognosis, and treatment response. The dysregulation of gene family members can serve as diagnostic indicators or therapeutic targets in specific cancer types. Additionally, understanding the functional implications of gene family alterations can provide insights into the underlying molecular processes driving tumor progression, allowing for the development of more targeted and effective therapies. With the advancement of multi-omics technologies, it has been revealed that there is a close relationship between gene families and the occurrence and development of tumors (Djos et al., 2012; Papaioannou, 2014; Chen et al., 2020; Wang Y. et al., 2021; Xie et al., 2022a; Keck et al., 2023).

Homeobox (HOX) genes represent the main subset of the homeobox family. These genes are evolutionarily highly conserved and regulate embryonic development and cell differentiation (Bhatlekar et al., 2018). HOX genes encode transcription factors that act as master regulators during embryogenesis processes, including apoptosis, receptor signaling, motility, and angiogenesis (Contarelli et al., 2020). A total of 39 human HOX family genes (HFGs) were distributed into four clusters (*HOXA*, *HOXB*, *HOXC*, and *HOXD*) according to their chromosomal localization (7p15, 17q21.2, 12q13, and 2q31, respectively). The HOXC genes encode a highly conserved family of transcription factors and play an important role in morphogenesis or development of neurons (Mendrzyk et al., 2006). Multiple studies have found that HFGs abnormal expression plays an essential role in cancer development (Bhatlekar et al., 2018; Li B. et al., 2019).

Most of the 39 HOX genes are aberrantly expressed in solid tumors, and their expression is frequently altered in cancer, including lung (Li L. et al., 2019), colon (Bhatlekar et al., 2019; Martinou et al., 2022), breast (de Bessa Garcia et al., 2020), pancreas (Kuo et al., 2019), prostate (Hatanaka et al., 2019), and ovarian cancers (Idaikkadar et al., 2019). Most HOX genes are expressed in the developing vertebrate central nervous system (CNS), where they play essential functions. Several studies have found that the expression pattern of HOX genes is dysregulated in gliomas (Abdel-Fattah et al., 2006; Costa et al., 2010). Previous studies have indicated that several members of HFGs are aberrantly expressed in pediatric gliomas. In 2010, Gaspar N, et al. discovered expression of *HOXA9*/*HOXA10* is regulated by demethylation mediated by the PI3-kinase pathway. Interestingly, inhibiting this demethylation process in combination with TMZ (temozolomide) treatment demonstrated a synergistic effect in a pediatric glioma cell line of KNS42. Furthermore, the research revealed that high levels of *HOXA9*/*HOXA10* gene expression were associated with a shorter survival in paediatric high grade glioma patient samples (Gaspar et al., 2010). Another research examined the expression of *HOXD* family genes by QPCR in 14 pediatric low-grade gliomas and found that *HOXD1* and *HOXD12* were overexpressed in tumor tissue compared to non-neoplastic tissues, while *HOXD3* presented lower expression in grade I glioma. HOXD8, D9, and D10 were found to be expressed in grade I gliomas, but not in non-neoplastic tissues. On the other hand, *HOXD4*, *D11*, and *D13* were not expressed in grade I gliomas (Buccoliero et al., 2009). However, the HFGs’ role in pediatric gliomas (PGs) remains unclear.

Due to the complex clinical and biological characteristics, improvements in PGs diagnosis and treatment are urgently needed. Molecular genetic analysis is essential for adequately classifying and monitoring biological behavior and clinical management of tumors. In this study, we classified PGs into two distinct subtypes based on the unsupervised consensus clustering of the HOX family genes (HFGs) transcriptome profiles of 571 PGs tumor samples. Each HOX-related subtype identified had distinct molecular features, such as molecular pathways, genomic alterations, immune checkpoints expression, and differences in patient survival. Furthermore, candidate drugs and potential targeted mechanisms were predicted for each PG subtype. We then explore the key HOX genes that played a crucial role in the PGs subtypes using random forest-supervised classification and the nearest shrunken centroid algorithm. Finally, we constructed a HOX-related signature to determine a PGs classification for utility in clinical practice.

## 2 Material and method

### 2.1 Public datasets preparation and normalization

Multiple levels of data, including whole-exome sequencing and mRNA-sequencing, along with complete survival, and clinical information, were obtained from several publicly available databases. The data of 486 PGs were downloaded from the Children’s Brain Tumor Tissue Consortium (CBTTC, https://cbttc.org/), 85 PGs from the International Cancer Genome Consortium (ICGC, https://dcc.icgc.org/), 163 PGs from Pediatric Brain Cancer (CPTAC/CHOP, Cell 2020; https://www.cbioportal.org/), and 53 PGs from Gene Expression Omnibus (GSE73038). We excluded 510 patients from CBTTC, 88 from ICGC, 55 from CPTAC, and 129 samples from GSE73038 that were not classified as gliomas or lacked prognostic/expression data, or had patients above 20 years of age at diagnosis. The detailed clinicopathological characteristics, including different grades of glioma patients, were summarized in Supplementary Table S1. The collected data underwent normalization, and the expression values were transformed using the logarithm. We used the “sva” algorithm to lessen the impact of the likely batch effects. The data of normal brain samples were obtained from the Genotype Tissue-Expression (GTEx) database. Among these four datasets, CBTTC and ICGC were selected to merge into a PGs cohort, which was then randomly divided into an training and an internal testing cohort. The other two datasets (CPTAC and GSE73038) were combined as an independent external validation cohort. Additionally, immunohistochemical (IHC) and multiple immunofluorescence (mIF) information was obtained from the Human Protein Atlas (HPA) database (https://www.proteinatlas.org/). In summary, this study involved comprehensive data collection from multiple sources, including genomic, clinical, and immunohistochemical information, to create robust cohorts for analysis and validation purposes.

### 2.2 Consensus clustering for different HOX-related subtypes

Since deregulated HOX gene expression has long been recognized as a driving force in tumorigenesis (Li B. et al., 2019), a total of 39 HFGs belonging to the four categories previously described were enrolled in our study (Supplementary Table S2). We applied an unsupervised clustering algorithm to explore a novel classification for PGs based on 39 HFGs expression matrix data to stratify those samples into different gene subtypes using the R package of ConsensusClusterPlus (Wilkerson and Hayes, 2010). A sampling of 80% of the data was used for the 1,000 iterations of the clustering procedure. The proportion of the ambiguous clustering algorithm, the consensus heatmap, and the relative change in the area under the cumulative distribution function (CDF) curve were used to determine the ideal number of clusters. Kaplan-Meier survival analysis was used to assess the associations between different clusters and overall survival. The expression profiles were standardized and principal component scores (PCA) were calculated. The PCA results were visualized in three dimensions using the R package “scatterplot3d” (Ligges and Mächler, 2002).

### 2.3 Somatic mutation and CNV analysis

Somatic mutation analysis was performed to identify the significantly mutated genes between different gene clusters using the R package “maftools” (Mayakonda et al., 2018). Waterfall plots were generated to display the mutation type and frequency of the top mutated genes in each cluster. The mutation type and frequency of the top mutated genes in each cluster were displayed by waterfall plots. The mutation data of 453 samples from PGs cohort are shown in Supplementary Table S3. We log-transformed the total mutation number to compare the mutation frequency differences between clusters and visualized them using R package “ggplot2” (The Wilcoxon test) (Wickham, 2016). We analyzed the copy number variations (CNVs) of different subtypes. The mean segment values were calculated by the log2 (cnv number/2) formula. Segment mean values > 0.2 was considered as a gain, while a value < -0.2 as a loss. The circos plots were used to display the CNV summary plots of each cluster using the R package “RCircos” (Zhang et al., 2013).

### 2.4 Gene set variation analysis and functional annotation

The R package “limma” (Ritchie et al., 2015) was utilized to identify differentially expressed genes (DEGs) between HOX-SI and HOX-SII subtypes. The screening criteria were *p*-value <0.01 and |log2 fold change (FC)| >1. To explore the functional implications of the DEGs, Gene Ontology (GO) and Kyoto encyclopedia of genes and genomes (KEGG) data sets in the molecular signature database (MsigDB) were obtained by the R package “msigdbr” (Version 7.2. 1) (Dolgalev, 2020). Furthermore, single-sample gene set enrichment analysis (ssGSEA) was performed using the R package “GSVA” (Hänzelmann et al., 2013) to quantify the difference in enrichment scores and pathways activity between the two subtypes in PGs. To further investigate the enrichment of pathways, the GSEA enrichment analysis was performed using the GSEA software (Version 4.2.3). Subsequently, the pathway activity score of ten oncogenic signaling pathways (Sanchez-Vega et al., 2018) in PGs subtypes was analyzed. The pathway activity score was calculated based on the method described by Han J et al. (Han et al., 2018). These scores were obtained by summing the normalized expression values of all genes contained in a pathway and then dividing by the square root of the number of genes of the signaling pathway. The genes associated with the ten oncogenic signaling pathways can be found in Supplementary Table S4.

### 2.5 Identification of the tumor immune infiltrating features of PGs

The composition and proportions of 22 different types of tumor-infiltrating immune cell fractions between subgroups were conducted using the “CIBERSORT” package (Newman et al., 2015) in PGs samples. Immune and stromal scores between subgroups were quantified based on the ESTIMATE algorithm in the R package “estimate” (Yoshihara et al., 2016) to assess tumor purity and applied R package “ggplot2” to show scoring differences between the two subgroups.

### 2.6 Immunotherapy and drug sensitivity prediction

Ye et al. summarized 34 immune checkpoint genes (Ye et al., 2020) in a reported study. However, in our study, the mRNA expression profile did not include VISTA. Therefore, the expression levels of 33 immune checkpoints, including well-known targets such as *CTLA4*, *PD-1*, *PD-L1*, and *PD-L2*, were screened to evaluate the sensitivity of immunotherapy between the two subtypes. The R package “oncoPredict” (Maeser et al., 2021) was used to predict the therapeutic response as measured by the half-maximal inhibitory concentration (IC50). The IC50 value reflects the sensitivity of a particular compound, with lower values indicating stronger sensitivity.

For further investigation of immunotherapy response, we downloaded the data of 298 urothelial cancer patients who received immunotherapy and detailed information about the response to PD-L1 blockade from the IMvigor210 datasets. Then, we used the IMvigor210 datasets to analyze the value of the HOX-related signature classifier in the predicted PD-1 response.

### 2.7 Development and verification of the HOX-based classifier via a random forest supervised classification algorithm

The random forest (RF) algorithm was applied to evaluate the contributions of 1008 DEGs that identified between HOX-SI and HOX-SII to clusters in PGs samples. Several iterative steps were performed, where one-third of the least essential DEGs were discarded at each step based on their importance score using the R package “ranger” (Wright and Ziegler, 2015). To ensure model stability, a total of 1000 decision trees were generated using the RF algorithm. Using a random forest-supervised classification algorithm, nine DEGs mostly related to the prognostic classification were selected among the initial 1008 DEGs based on their important permutation score. According to combinations of the nine DEGs, 511 (2^9^–1) combinations were obtained. For each combination, a signature was developed using the nearest shrunken centroid algorithm and Euclidean distance in the training set. Two centroids, representing “high-risk” and “low-risk” groups, were created based on the mean gene expression profiles of the DEGs in patients with good prognosis and those with poor prognosis, respectively. The euclid distances between all samples and the two centroids were calculated.

Subsequently, a prognostic classifier were developed for all combinations (N = 2^9^–1 = 511) of 9 HOX-related genes using the nearest shrunken centroid algorithm. Dic is the Euclid distance between the mean expression profile of the HOX-related genes combination and the two centroids. The Euclid distances (Dic_i_) classify sample i into HOX-SI or HOX-SII. After the analysis, a signature containing of five HOX-related genes was selected as the optimal signature. The following formula was used to calculate the Dic_i_, where n is the number of HOX-related gene signature, Exp_j_ is the gene expression value of the signature genes, and CD is the centroids of “high-risk” or “low-risk” groups:

$${Dic}_{i}=\sqrt{\sum_{j}^{n}\left({\mathrm{Exp}}_{j}-CD\right)}$$

### 2.8 Single-cell RNA-seq (scRNA-seq) analysis

The single-cell RNA-sequencing data of 13 pediatric medulloblastomas, along with their corresponding clinical information, were downloaded from the GEO database (GSE119926). This dataset allows for detailed cell type annotation at the single-cell level. Based on the R package “Seurat”, the Uniform Manifold Approximation and Projection (UMAP) plot was used to visualize the distribution and expression of *HOXC4*, *HOXC5*, and *HOXC6* in the 13 pediatric medulloblastoma different cell types and four molecular subgroups of medulloblastoma.

### 2.9 Statistical analysis

In this study, all analyses were conducted by R software (version 4.1.2, Institute for Statistics and Mathematics, Vienna, Austria 4). Wilcoxon test was conducted for the comparisons between the two groups. The chi-square test examined the relationships between glioma subgroups and clinical characteristics. Differences in survival were analyzed by the Kaplan-Meier method, and significance was determined by the log-rank test. Univariate and multivariate analysis was done using the multivariate Cox proportional hazard regression model. All tests were two-sided, and a *p*-value of less than 0.05 was considered significant.

## 3 Results

### 3.1 Genetic and transcriptional profile of HFGs in pediatric gliomas

**FIGURE 1:**
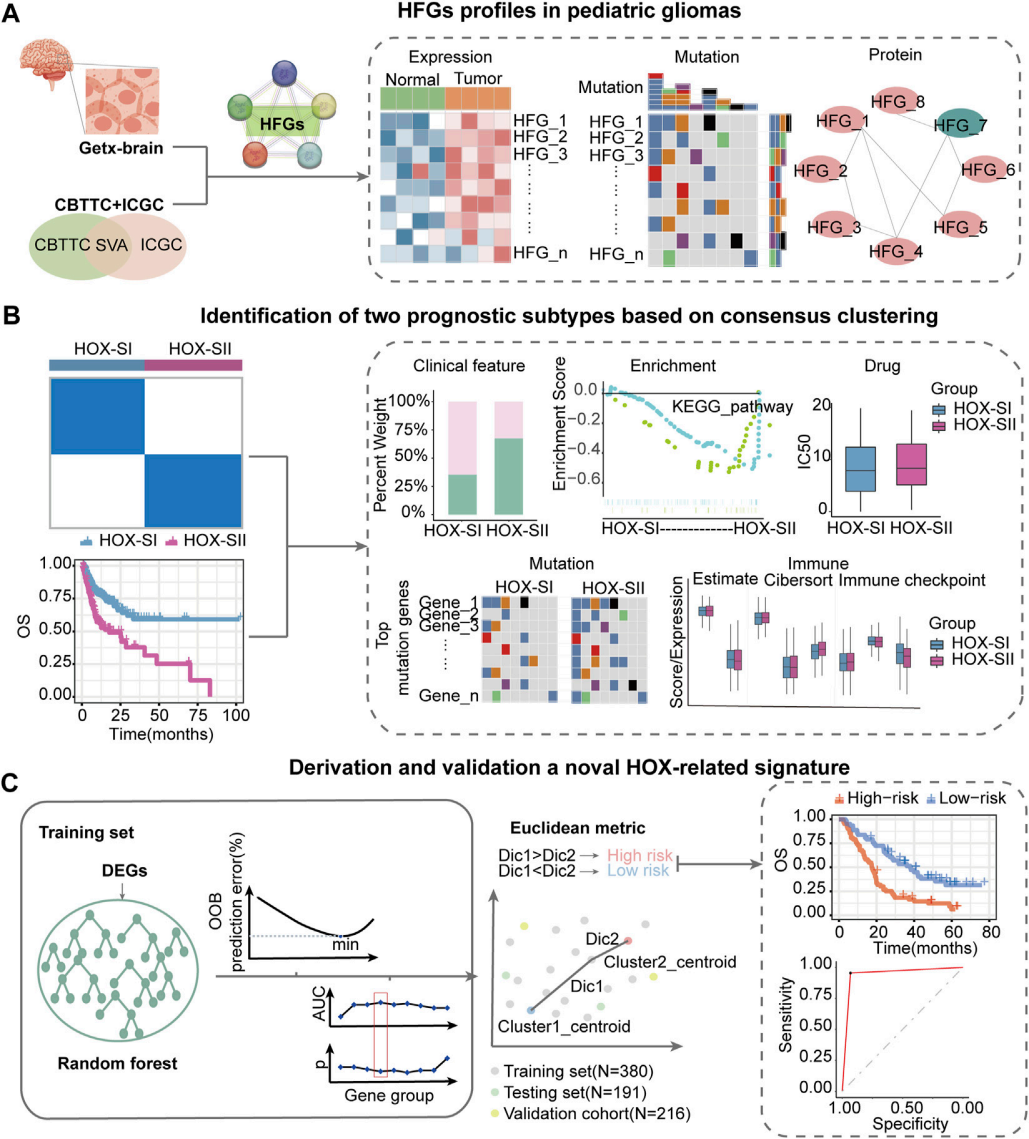
Workflow of data analysis in our study. **(A)** HFGs profiles in pediatric gliomas. We will analyze the expression patterns, mutation characters, and their protein network relationship of HFGs in PGs samples. **(B)** Identification of two prognosis subtypes based on consensus clustering. We will classify pediatric gliomas into two subtypes, namely HOX-SI and HOX-SII, based on the expression patterns of the HFGs. We will explore the clinical characteristics, signaling pathways, drug sensitivity, gene mutation patterns, and immune microenvironment differences between these two subtypes. **(C)** Derivation and validation a novel HOX-related signature. We will construct a diagnostic and prognostic model based on the differential expression of genes between the HOX SI and HOX SII subtypes. Using the random forest method, we identify key DEGs that contribute significantly to the classification of subtypes. Then, we employ the Euclidean distance algorithm to develop a novel HOX-related signature for diagnosing and predicting the prognosis of PGs patients based on the high or low-risk classification. HFGs, Homeobox family genes; PGs, pediatric gliomas; Dic, distance; OOB, out-of-bag; OS, overall survival; AUC, area under curve.

**FIGURE 2:**
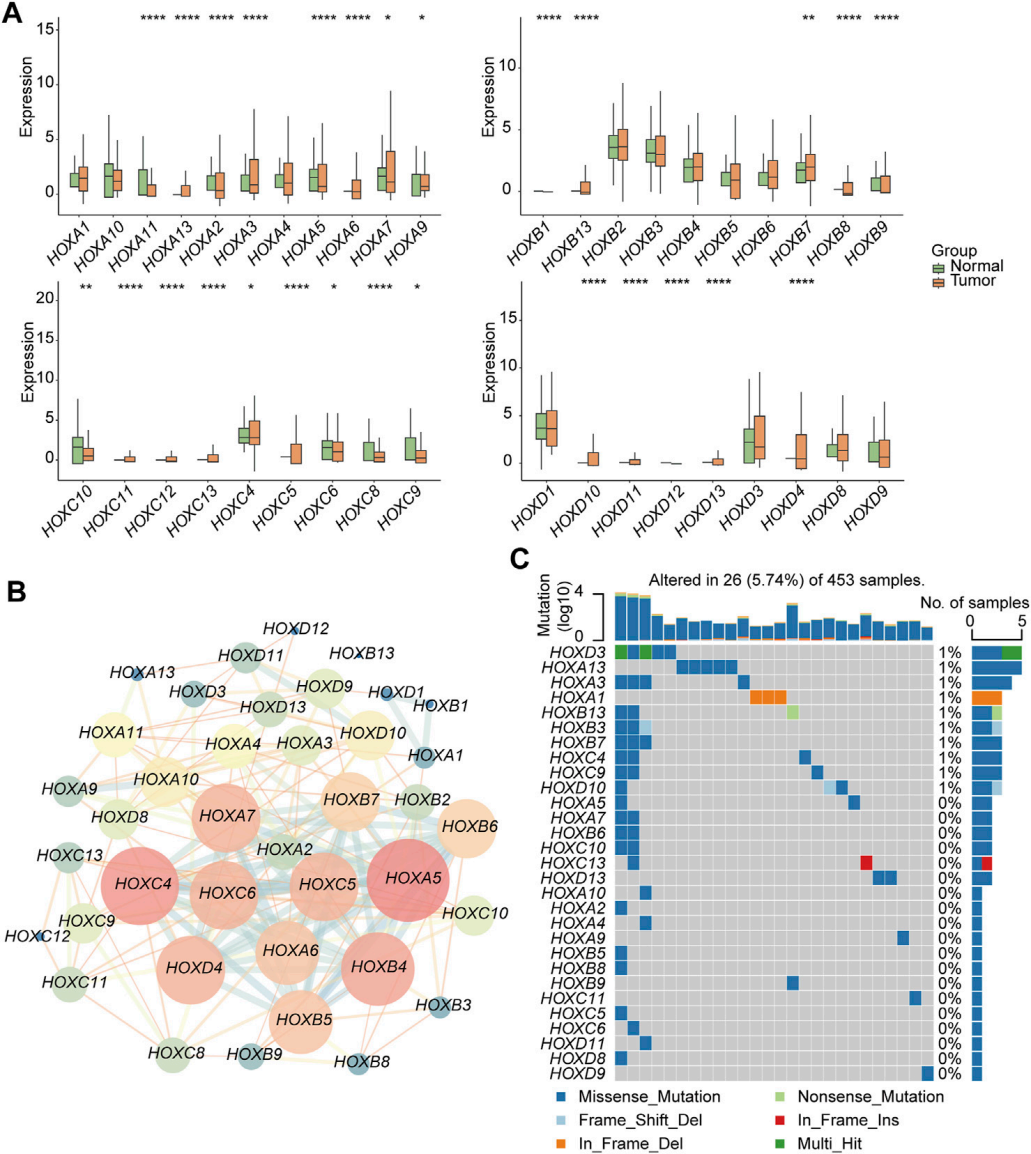
Genetic and transcriptional alterations of HFGs in pediatric gliomas (PG). **(A)** Box plots showed the differences in expression of *HOXA*, *HOXB*, *HOXC*, and *HOXD* genes in normal and tumor tissues respectively. **(B)** The correlations among 39 HFGs. Pink circles indicate a higher degree. Blue circles indicate a lower degree. Circle size represents the combined score. The higher combined score, the larger circle size. **(C)** The mutation frequency of 39 HFGs in 453 patients with PGs from the CBTTC + ICGC cohort. Mutation frequency (%) was derived from the number of mutation samples/the total number of samples (N = 453). *p < 0.05, **p < 0.01; ***p < 0.001.

This workflow of our study consists of three main parts. In the first part, we aim to investigate the specific features of the HFGs in PGs. We will analyze the expression patterns, mutation characters, and their protein network relationship of HFGs in PGs samples (Figure 1A). In the second part, we classify PGs into two subtypes, namely HOX-SI and HOX-SII, based on the expression patterns of the HFGs. We will explore the clinical characteristics, signaling pathways, drug sensitivity, gene mutation patterns, and immune microenvironment differences between these two subtypes. By understanding the distinct features of each subtype, we aim to uncover potential prognostic markers and therapeutic targets specific to each subtype of PGs (Figure 1B). In the third part, we will construct a diagnostic and prognostic model based on the differential expression of genes between the HOX SI and HOX SII subtypes. Using the random forest method, we will identify the key DEGs that contribute significantly to the classification of subtypes. Additionally, we employ the Euclidean distance algorithm to develop a novel HOX-related signature for diagnosing and predicting the prognosis of PGs patients based on the high or low-risk classification (Figure 1C). We selected 39 HFGs for analysis, and their detailed information can be found in Supplementary Table S2. To reveal the expression levels of 39 HFGs in PGs, we compared their expressions in tumor and normal tissues. Compared with the normal tissues, most of the HOX genes showed increased expression in tumor tissues, only *HOXB1* and *HOXD1* decreased expression in PGs samples, and the difference in expression of these HFGs in normal and tumor tissues was statistically significant except *HOXB8* and *HOXC12* (Figure 2A). Since HFGs functions are interconnected (Bhatlekar et al., 2014a), we constructed a protein-protein interaction (PPI) network to visualize the relationships among these genes. In PPI, several hub genes were identified including HOXA5, HOXA6, HOXA7, HOXB4, HOXB5, HOXB6, HOXB7, HOXC4, HOXC5, HOXC6, and HOXD4 (Figure 2B). These hub genes play important roles in the interaction network of HFGs. Furthermore, we assessed the association between HFGs expression and overall survival (OS). Most HFGs exhibited significant differential transcriptional expression between tumor and normal tissues and were significantly correlated with OS (Supplementary Table S5). This suggests that HFGs abnormal expression may play a crucial role in the pathogenesis and progression of PGs. Additionally, we analyzed somatic alterations of 39 HFGs in PGs cohort. The mutation frequency of these genes was found to be low, with only 26 out of 453 PGs patients (4.55%) showing HFGs mutations. The landscape of HFGs mutations in the 26 PGs patients were present in Figure 2C.

### 3.2 Identification of potential subtypes in PGs based on HFGs

**FIGURE 3:**
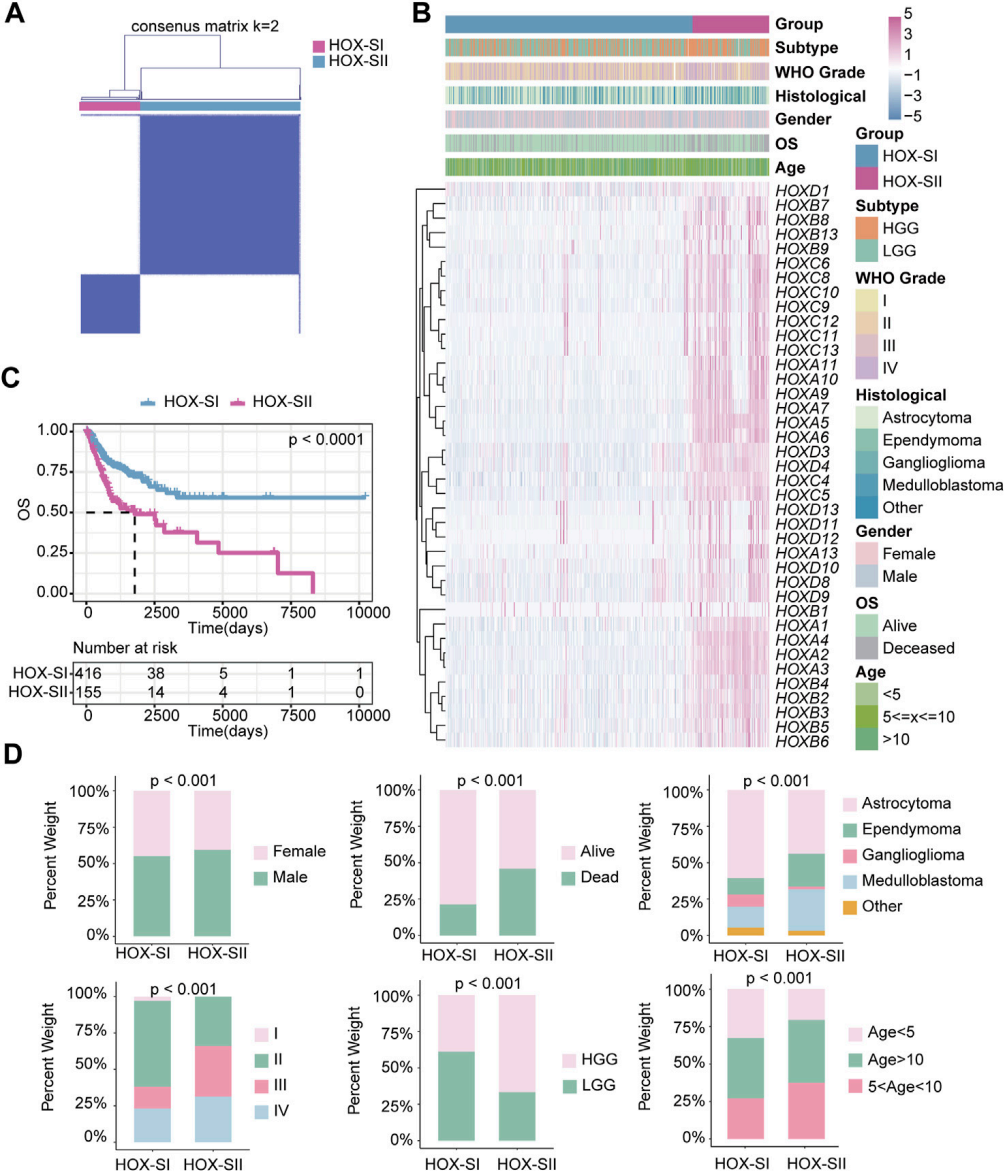
The clinical values in HOX-regulated gene subgroups in pediatric glioma patients based on consensus clustering. **(A)** Consensus matrix of PGs cohorts for K = 2. **(B)** Heatmap demonstrates the expression levels of HFGs in different subtypes and the distribution of the clinical features. Purple indicates higher gene expression, and blue indicates lower gene expression. **(C)** Kaplan-Meier curves showed the OS difference between HOX-SI and HOX-SII. **(D)** Comparisons of clinicopathological variables between tumors of the two clusters in the PGs cohort. p< 0.05 was considered statistically significant.

First, the 39 HFGs expression profile matrix of 571 PGs samples was generated and normalized by R package “sva.” Then, unsupervised consensus clustering of the HFGs was performed. The 571 PGs from the PGs cohort were divided into two clusters (Figure 3A, Supplementary Figure S1A,B): HOX-subtype I (HOX-SI) and HOX-subtype II (HOX-SII). The PCA showed that HOX-SI and HOX-SII could be distinguished based on this classification (Additional file 2: Supplementary Figure S1C). The gene expression profile and clinicopathological parameters, including age at diagnosis, gender, tumor stage (WHO I–IV), histology type, and glioma subtype, are illustrated in a heatmap. The expression level of HFGs had significantly different between the two clusters. Most genes showed higher expression levels in HOX-SII (Figure 3B). The Kaplan-Meier survival analysis indicated that patients in HOX-SI subgroup showed significantly better OS than those in HOX-SII (*p* < 0.0001, Figure 3C). These findings indicate that PGs of different subtypes were correlated with distinct clinical outcomes, with HOX-SII exhibiting a worse prognosis and clinical features. Moreover, significant differences were observed in the clinicopathological characteristics between HOX-SI and HOX-SII. Patients with HOX-SII tumors were diagnosed at an older age, had a higher proportion of males, a higher mortality rate, a higher occurrence of ependymoma and medulloblastoma, higher WHO grades, and a higher frequency of HGG compared to those in the HOX-SI group (Figure 3D).

### 3.3 Somatic mutations and CNVs characteristic in PGs clusters

**FIGURE 4:**
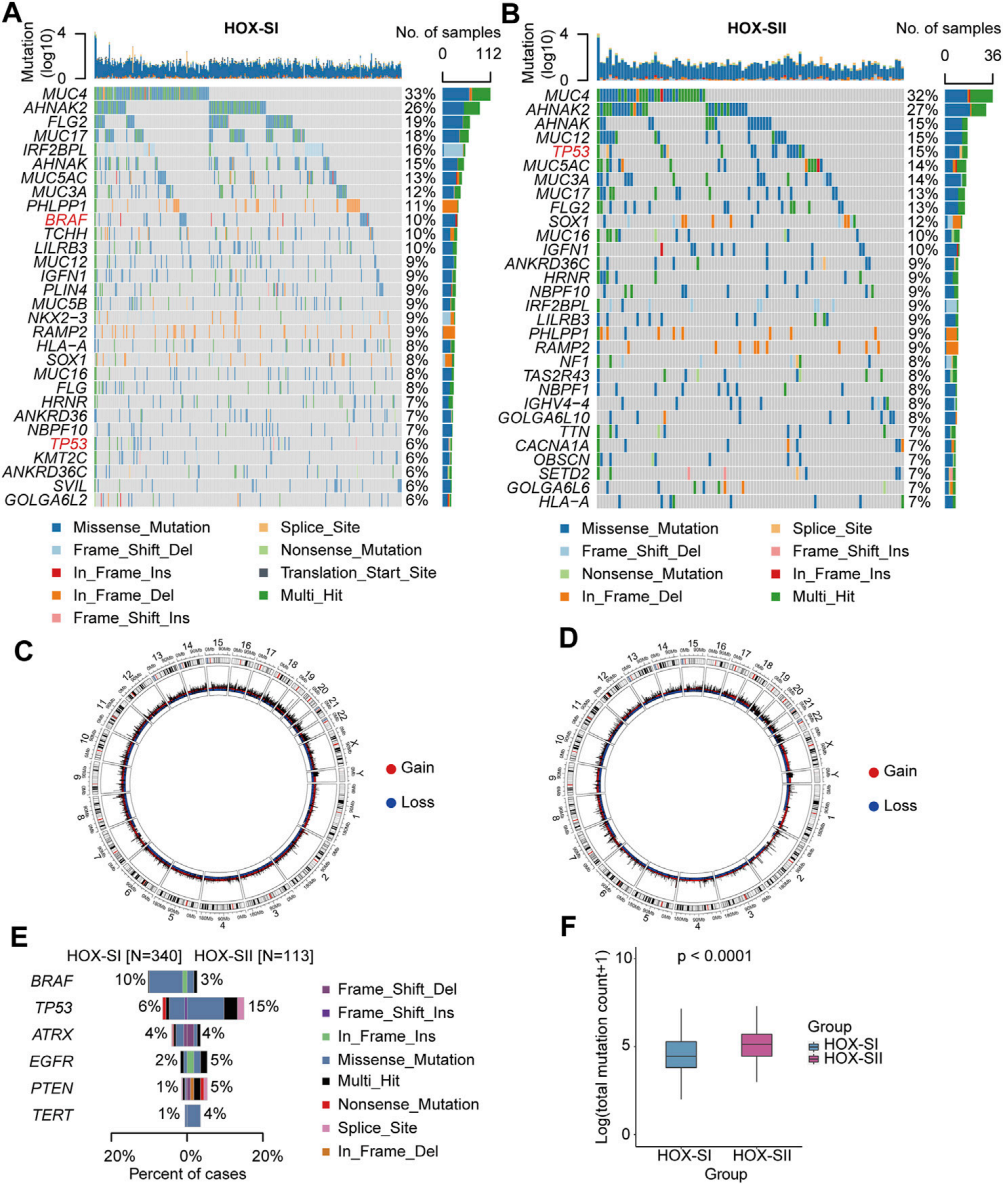
Somatic variations of the two subtypes. **(A, B)** Waterfall plots showing the top 30 mutated genes of HOX-SI **(A)** and HOX-SII **(B)**. The genes highlighted in red are significantly differentially mutated genes between the HOX-SI and HOX-SII groups. **(C, D)** Circos plots of HOX-SI **(C)** and HOX-SII **(D)** subtypes reveal CNV of chromosomes, with red dots representing gains, blue dots representing losses, and black dots representing no significant CNA. **(E, F)** Comparison of the commonly altered genes **(E)** and total mutation counts **(F)** between HOX-SI and HOX-SII in PGs. *p*-value was inferred from Wilcoxon test.

The top mutated genes in HOX-SI and HOX-SII are shown in Figures 4A,B. *MUC4*, *AHNAK2*, *AHNAK*, *FLG2*, *MUC5AC*, *MUC3A*, and *MUC17* were the most common alterations in PGs (Additional file 1: Supplementary Table S6). The CNVs were analyzed in PGs subtypes. The results showed that the CNVs between the two subgroups had no significant difference (Figures 4C,D, Additional file 1: Supplementary Table S7, Additional file 2; Supplementary Table S1D). Subsequently, we compare the commonly altered genes in PGs between HOX-SI and HOX-SII. The mutation frequency of *BRAF* was higher in HOX-SI, whereas *TP53, EGFR, PTEN,* and *TERT* were higher in HOX-SII (Figure 4E). Patients with HOX-SII exhibited a higher total mutation count than HOX-SI (*p* < 0.001, Figure 4F).

### 3.4 Biological properties and molecular mechanism of the two PGs subtypes

**FIGURE 5:**
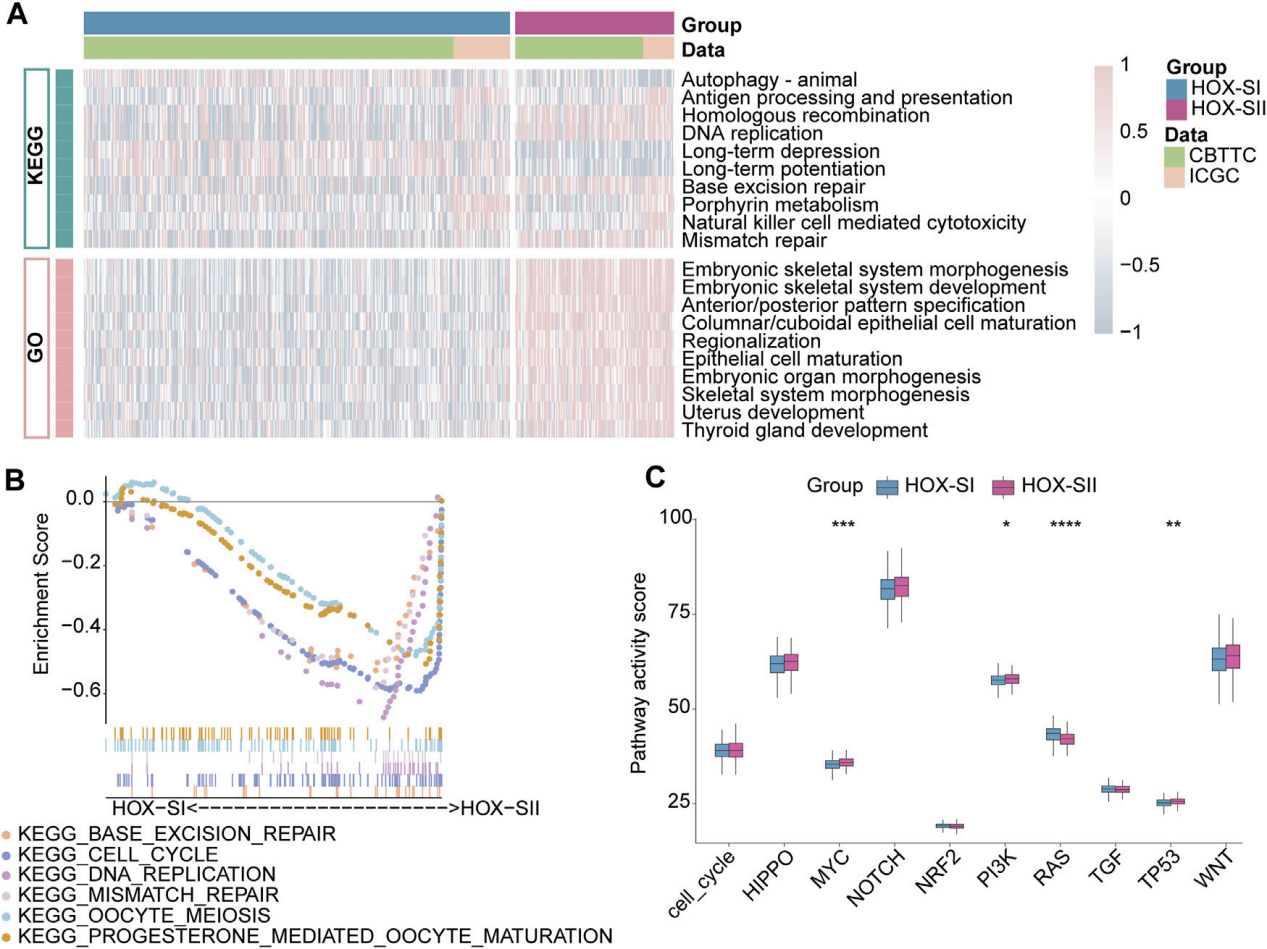
Functional annotations of the two PGs subtypes. **(A)** GO-related and KEGG-related GSVA show the activation status of biological behaviors in HOX-SI and HOX-SII in PGs. Pink showed upregulated pathways, and blue showed downregulated pathways. **(B)** Multiple malignant regulatory pathways were significantly enriched in HOX-SII by GSEA analysis. **(C)** The differences of ten canonical signaling pathways between two PGs subtypes. GO, Gene Ontology; KEGG, Kyoto Encyclopedia of Genes and Genomes; GSVA, gene set variation analysis. *p < 0.05, **p < 0.01, ***p < 0.001, ****p < 0.0001. p< 0.05 was considered statistically significant.

To explore the pathways and molecular mechanisms correlated to the PGs prognosis classifications, GO- and KEGG-related gene set variation analysis (GSVA) was performed in the PGs cohort, including CBTTC and ICGC datasets. The results revealed tumor-associated DNA damage and cell differentiation-related signal pathways were significantly enriched in HOX-SII (Figure 5A, Additional file 1: Supplementary Table S8). Furthermore, the pathways such as progesterone-mediated oocyte maturation, cell cycle, DNA replication, oocyte meiosis, mismatch repair, and base excision repair pathways were significantly enriched in HOX-SII (Figure 5B). We further analyzed the activity of ten oncogenic signaling pathways in PGs subtypes using RNA expression data. Compared with subtype HOX-SI, HOX-SII showed higher pathway activity scores, particularly in the MYC (*p* < 0.001), PI3K (*p* = 0.019), and TP53 (*p* = 0. 002) pathways, but lower scores of RAS (*p* < 0.001) pathway (Figure 5C, Additional file 1: Supplementary Table S9).

### 3.5 Different immune cell infiltration profiles in PGs subtypes

**FIGURE 6:**
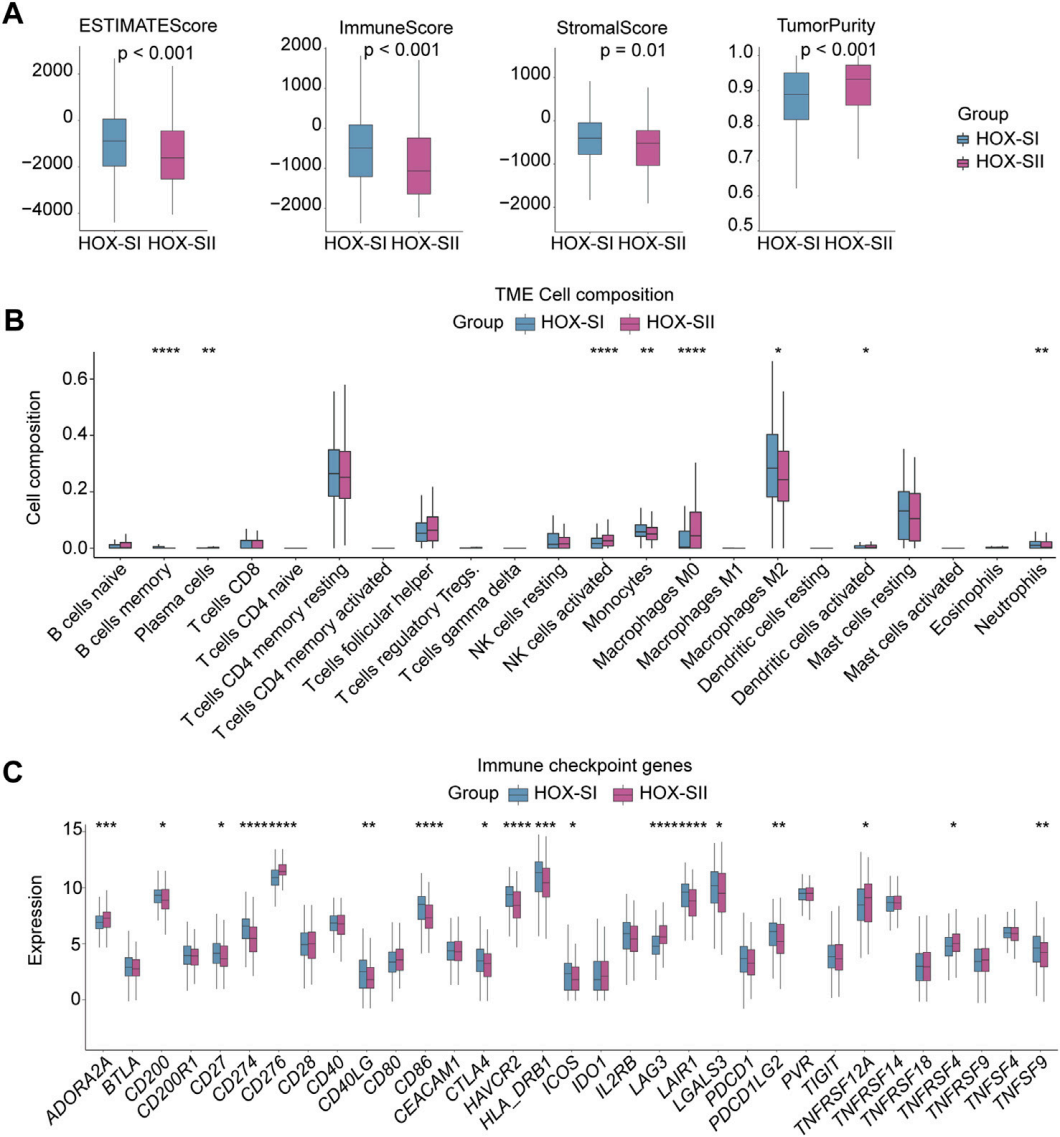
Tumor microenvironment features of the two PGs subtypes. **(A)** Comparisons of tumor immune, stromal, and ESTIMATE scores between two subtypes in the PGs cohort. **(B)** Comparison of the infiltration of 22 types of immune cells between two clusters. **(C)** Distinct expression of 33 immune checkpoints between HOX-SI and HOX-SII in the PGs. *p < 0.05, **p < 0.01; ***p < 0.001, ****p < 0.0001. p< 0.05 was considered statistically significant.

Immunity and stromal scores calculated based on the ESTIMATE were performed to explore the composition of the tumor microenvironment (TME) in PGs. The immune score (*p* < 0.001), stromal score (*p* = 0.010), and ESTIMATEscore (*p* < 0.001) were significantly higher in HOX-SI than HOX-SII. This indicates a higher abundance of immune and stromal cells and a lower proportion of tumor purity (*p* < 0.001) in PGs with HOX-SI tumors than in HOX-SII tumors (Figure 6A). Then, the abundance of 22 types of tumor-infiltrating immune cells was evaluated in PGs classifications using CIBERSORT. CD4 memory resting T cells, M2 Macrophages, and resting mast cells were identified as the most common immune cells in the TME of PGs. The majority of tumor-infiltrating immune cells were more abundant in HOX-SI tumors than in HOX-SII. Several types of immune cells, including M2 Macrophages, Memory B cells, Monocytes, and neutrophils, exhibited significantly higher abundance in HOX-SI. Conversely, plasma cells, activated NK cells, M0 Macrophages, and activated dendritic cells were more abundant in HOX-SII (Figure 6B). Among 33 immune checkpoints (ICs), 18 ICs demonstrated significantly different expression levels in PGs subtypes. Most ICs showed higher expression levels in HOX-SI tumors, including *CD200* (*p* = 0.018), *CD27* (*p* = 0.018), *CD274* (PDL1, *p* < 0.001), *CD40LG* (*p* = 0.002), *CD86* (*p* < 0.001), *CTLA4* (*p* = 0.038), *HAVCR2* (*p* < 0.001), *HLA_DRB1* (*p* < 0.001), *ICOS* (*p* = 0.010), *LAIR1* (*p* < 0.001), *LGALS3* (*p* = 0.019), *PDCD1* (PD1, *p* = 0.073), *PDCD1LG2* (PDL2, *p* = 0.010), and *TNFSF9* (*p* = 0.004) (Figure 6C). The findings suggest that PGs patients in the HOX-SI subtype may obtain a better response to immune checkpoint inhibitors (ICI) administration.

### 3.6 Targeted therapeutic sensitivities prediction

**FIGURE 7:**
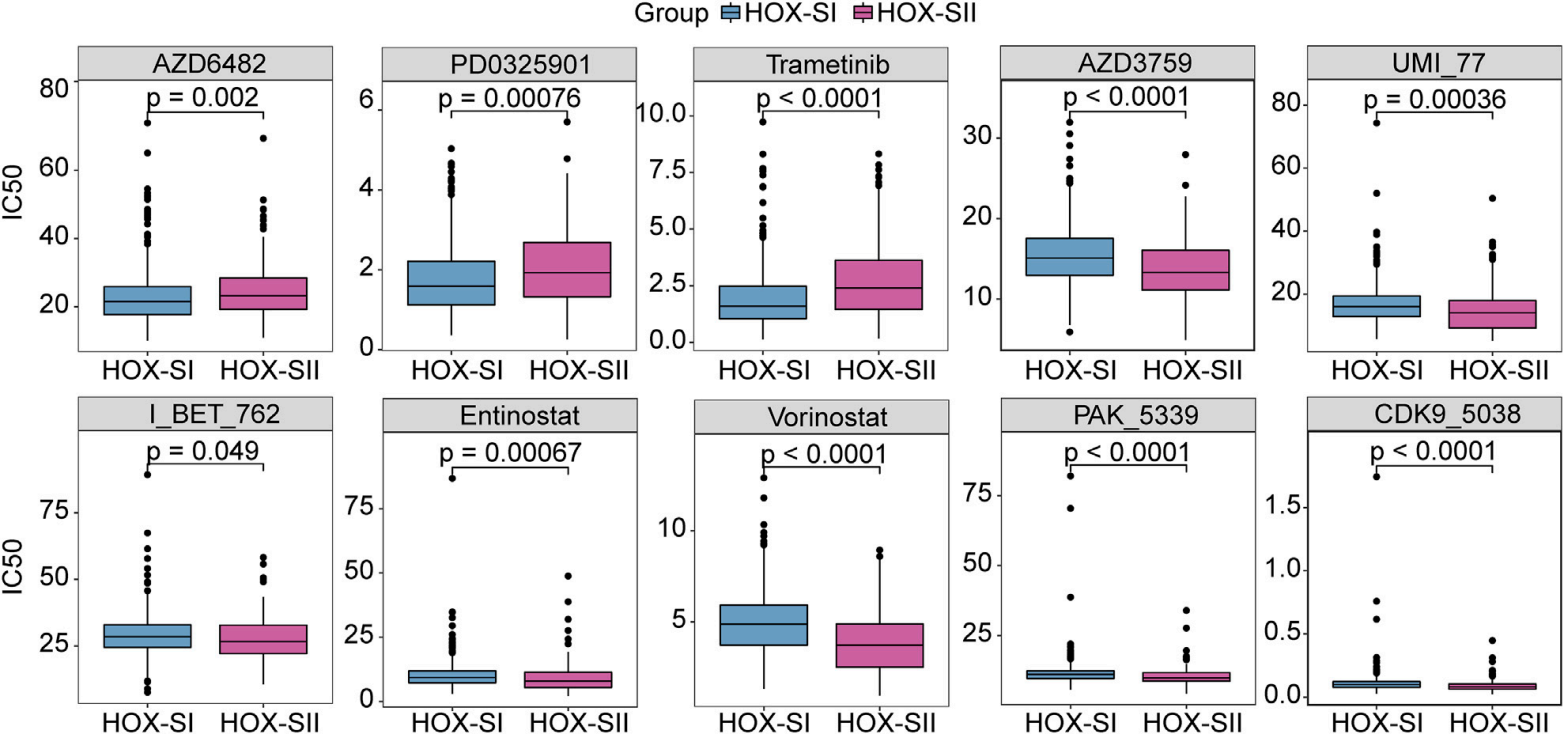
The drug sensitivity prediction in HOX-SI and HOX-SII. In AZD6482, PD0325901, and Trametinib, the median IC50 of HOX-SI was significantly lower than those of HOX-SII. In AZD3759, UMI_77, I_BT_762, Entinostat, Vorinostat, and PAK_5339, HOX-SII had a significantly lower median IC50 than HOX-SI. Gene expression and drug sensitivity information of cancer cell lines were obtained from The Genomics of Drug Sensitivity in Cancer (GDSC) database. IC50, half maximal inhibitory concentration.

By compiling IC50 values for each sample from the Genomics of Drug Sensitivity in Cancer database, we used the pRRophetic algorithm to determine which drugs may be effective for glioma patients. Subsequently, we analyzed multiple targeted compound sensitivities in PGs subtypes. Ultimately, ten compounds were obtained based on significant differences in predicted IC50 values between the two subtype groups, with the HOX-SI group was more sensitive to most compounds. The ten drugs that necessitate further study in PGs, including Zorifertinib (AZD3759), a PI3Kβ inhibitor (AZD6482), a CDK9 inhibitor (CDK9_5038), two histone deacetylase inhibitor (Entinostat and Vorinostat), Mcl-1 inhibitor (UMI_77), a BET inhibitor (I_BT_762), a PAK inhibitor (PAK_5339), and two MEK inhibitor (PD0325901 and Trametinib). Analysis of drug sensitivity showed that patients in the HOX-SI group were predicted to be more sensitive to AZD6482, PD0325901, and Trametinib compared to those in the HOX-SII group (Figure 7). In contrast, patients in the HOX-SII group were predicted to be more sensitive to AZD3759, UMI_77, I_BT_762, Entinostat, Vorinostat, and PAK_5339 compared to those in the HOX-SI group (Figure 7).

### 3.7 Derivation of a HOX-related diagnosis and prognostic signature in PGs

**FIGURE 8:**
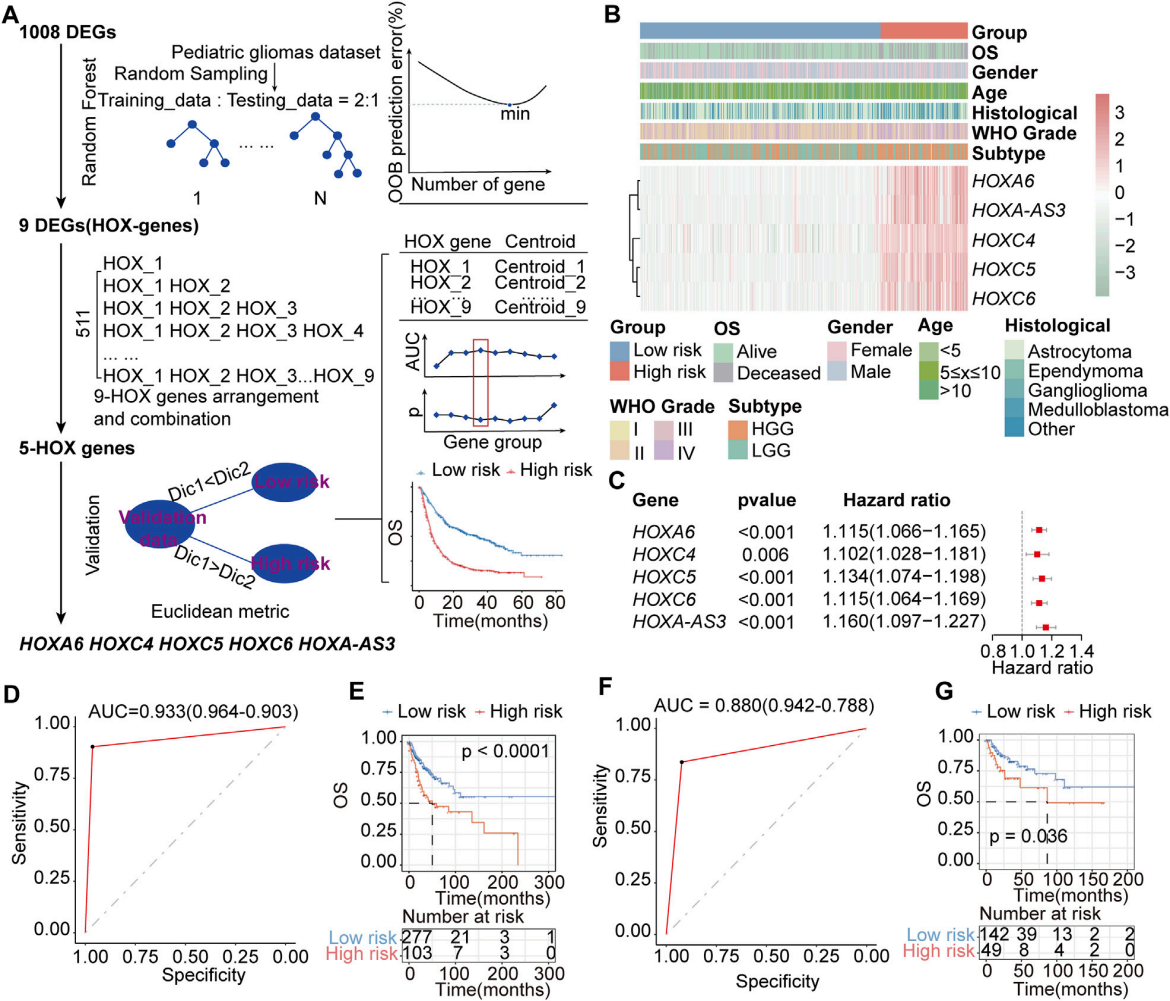
Construction of the HOX-related classifier signature. **(A)** The workflow of identifying the HOX-hub-gene signature in the training set. **(B)** Expression heatmap of three core HOX genes and integrated results of HOX-related subtypes and clinical features. Red indicates higher gene expression, and green indicates lower gene expression. **(C)** Univariate Cox regression analysis of HOX-related signature genes. Hazard ratio>1 indicates that the gene is a risk factor, and hazard ratio<1 indicates that the gene is a protective factor. p< 0.05 was considered statistically significant. **(D, E)** ROC curves of the HOX-related Subtype Classifier in distinguishing two subtypes in the training set. **(D)** and interval testing set **(E)**. **(F, G)** Kaplan-Meier OS curves for the two groups in training **(F)** and interval testing sets **(G)**. *p*-value was inferred from the log-rank test.

We aimed to screen core genes relevant to PGs subtypes based on 1008 DEGs (Additional file 1: Supplementary Table S10) between HOX-SI and HOX-SII to build a clinically applicable classifier that could conveniently predict the HOX-related prognostic subtypes of PGs patients. To avoid overfitting and eliminate noise in data, 571 PGs patients in the PGs cohort were randomly divided into training (n = 380) and testing (n = 191) groups with a 2:1 ratio. The RF algorithms were used based on the training set. The analysis details showed in the flow chart in Figure 8A. Nine DEGs mostly related to prognostic/diagnostic classification were chosen from 1008 DEGs based on the important permutation score using the random forest supervised classification algorithm. The diagnostic model was subsequently established using the nearest shrunken centroid algorithm.

We next explored the association between HFGs expression and the OS of patients with PGs. Ultimately, a HOX-related signature consisting of *HOXA6*, *HOXC4*, *HOXC5*, *HOXC6*, and *HOXA-AS3* was selected from the training set, considering a balance between accuracy and the number of HFGs (Figures 8B,C). In this signature, the ‘low-risk’ and ‘high-risk’ centroids were determined as (0.61, 4.55, 0.74, 1.62, 0.43) and (5.70, 8.02, 4.93, 5.52, 4.46), representing the average expression level of the five HFGs for the patients with good and poor prognosis, respectively. The signature was defined as follows:

$${Dic}_{i,1}=\sqrt{{\left({\mathrm{Exp}}_{HOXA6}^{i}-0.61\right)}^{2}+{\left.{\left(\mathrm{Exp}\right.}_{HOXC4}^{i}-4.55\right)}^{2}+{\left({\mathrm{Exp}}_{HOXC5}^{i}-0.74\right)}^{2}+{\left({\mathrm{Exp}}_{HOXC6}^{i}-1.62\right)}^{2}+{\left({\mathrm{Exp}}_{HOXA-AS3}^{i}-0.43\right)}^{2}}$$

$${Dic}_{i,2}=\sqrt{{\left({\mathrm{Exp}}_{HOXA6}^{i}-5.70\right)}^{2}+{\left.{\left(\mathrm{Exp}\right.}_{HOXC4}^{i}-8.02\right)}^{2}+{\left({\mathrm{Exp}}_{HOXC5}^{i}-4.99\right)}^{2}+{\left({\mathrm{Exp}}_{HOXC6}^{i}-5.22\right)}^{2}+{\left({\mathrm{Exp}}_{HOXA-AS3}^{i}-5.52\right)}^{2}}$$

The ${\mathrm{Exp}}_{HOXA6}^{i}$ , ${\mathrm{Exp}}_{HOXC4}^{i}$ , ${\mathrm{Exp}}_{HOXC5}^{i}$ ${\mathrm{Exp}}_{HOXC6}^{i}$ , and ${\mathrm{Exp}}_{HOXA-AS3}^{i}$ denoted as the expression level of *HOXA6*, *HOXC4*, *HOXC5*, *HOXC6*, and *HOXA-AS3* for sample i, respectively. A patient was classified into the ‘low-risk’ group if ${Dic}_{i,1}$ < ${Dic}_{i,2}$ according to the patient’s five hub genes expression values and into the ‘high-risk’ group if not. The euclidean distances of each sample are shown in Supplementary Table S11.

The receiver operating characteristic (ROC) curve demonstrated that this classifier was reliable, with an area under curve (AUC) of 0.933 (Figure 8D) in the training set and an AUC of 0.880 (Figure 8F) in the internal testing set. The prognostic value of the HOX-related signature was evaluated by log-rank test in both the training and testing sets. In the training group, patients were divided into a high-risk group (n = 103) or a low-risk group (n = 277) based on the HOX-related signature. Patients with the high-risk signature exhibited significantly shorter OS than those with the low-risk signature (median OS: NR vs 50.3 months, HR:2.218, 95% CI: 1.515-3.245, *p* < 0.0001, Figure 8E). Similarly, in the testing, patients were classified as high-risk (n = 49) or low-risk (n = 142) according to their HOX-related signature (median OS: NR vs 86.5 months, HR:1.957, 95% CI: 1.031-3.714, *p* = 0.036, Figure 8G).

Cox regression analysis was performed to assess the impact of age, gender, histological type, WHO grade, stage subtype, and the HOX-related signature. The results from the training set showed that the high-risk HOX-related signature (HR:1.769, 95% CI: 1.174-2.665, *p* = 0.006) and WHO grade (HR: 1.547, 95% CI: 1.233-1.941, *p* < 0.001) were significantly correlated with poor OS in PGs patients Supplementary Table S12. The testing set showed that the HOX-related signature (HR:2.113, 95% CI: 1.179-3.789, *p* = 0.012) and WHO grade (HR:1.519, 95% CI: 1.112-2.076, *p* = 0.009) were identified as independent prognostic factors for PGs patients Supplementary Table S12. Thus, the multivariable Cox regression analysis revealed that the HOX-related signature had a good predictive ability for PGs patient survival, independent of other clinical-pathological factors.

### 3.8 Prediction of the prognostic performance and immunotherapy response of the HOX-related signature

**FIGURE 9:**
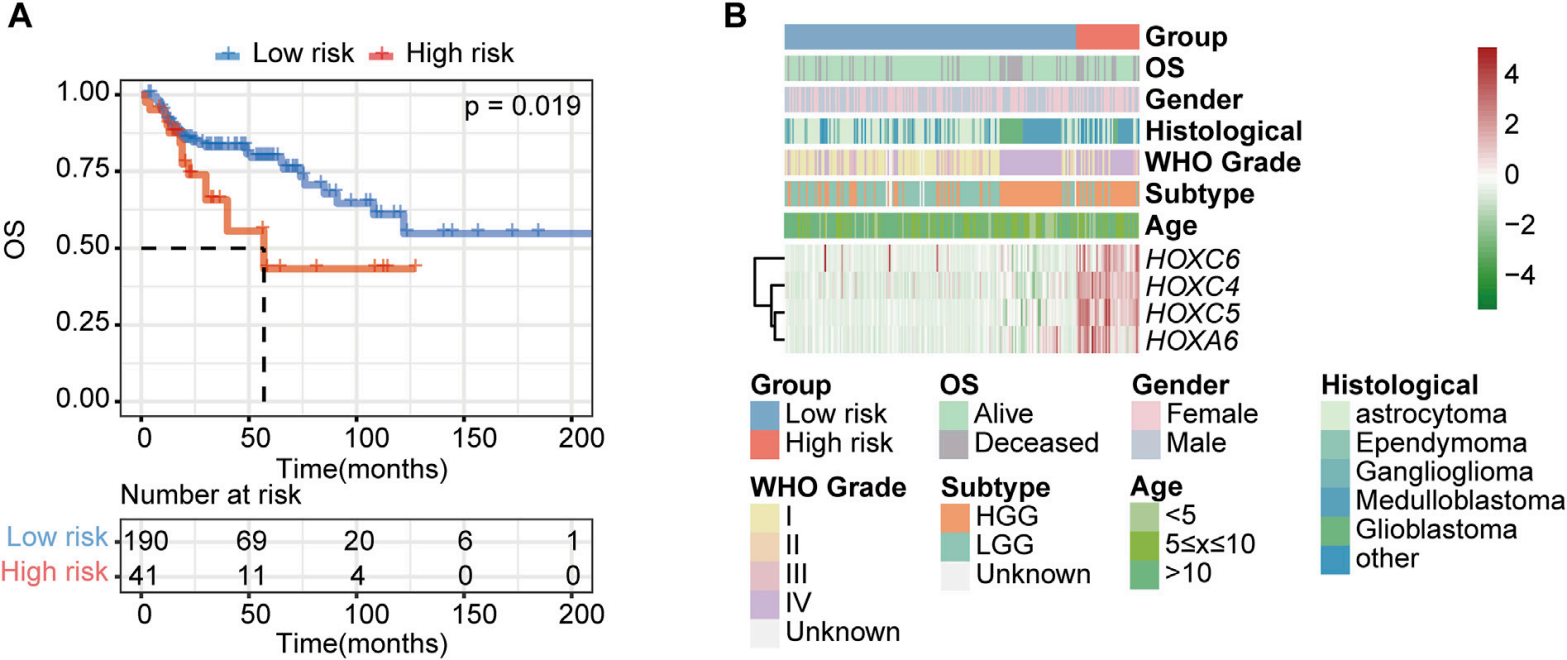
Validation of the HOX-related classifier signature in an external independent cohort. **(A)** Kaplan-Meier curves showed the OS difference between high- and low-risk groups in independent external validated cohort (CPTAC and GSE73038). *p*-value was inferred from log-rank test. **(B)** Expression heatmap of HOX-related signature genes and integrated results of HOX-related subtypes and clinical features. Red indicates higher gene expression, and green indicates lower gene expression.

To further validate the HOX-related subtype classifier, another two gliomas datasets (CPTAC and GSE73038) were merged into a new independent cohort with 231 PGs patients enrolled. Using the same classifier formula, we calculated the distance value again for each patient in an independent external validated cohort based on the expression values of the HOX-related signature genes and their corresponding survival data. Then we divided the patients into two subgroups based on the same classifier threshold. The validated cohort consisted of 41 patients with high risk and 190 patients with low risk. The Kaplan-Meier curve confirmed that patients in the high-risk group had a worse OS than those in the low-risk (Figure 9A), which is consistent with the findings in the PGs cohort, implying that this HOX-related classifier signature was an independent prognostic factor in PGs. Additionally, the expression levels of the hub genes were also found to be increased in the high-risk group of tumors in this independent external validated cohort (Figure 9B).

To estimate the predictive ability of the HOX signature for immunotherapy response, 298 patients who received PDL1 blockade treatment from IMvigor210 were enrolled. The results showed that patients in the HOX-SII group exhibited poor prognosis (Supplementary Figure S2A) and a much lower response rate to immunotherapy compared to those in the HOX-SI group (Supplementary Figure S2B).

### 3.9 Validation of the HOX-related signature genes in single-cell and HPA database

The consensus clustering analysis revealed that the HOX-SII subtype had a higher proportion of medulloblastoma samples. To explore the relationship between this phenomenon and HOX gene expression, we downloaded the single-cell data of 13 samples of pediatric medulloblastoma. The annotation results indicated the samples mainly consisted of neurons, neuroepithelial cells, astrocytes, and smooth muscle cells (Supplementary Figure S3A). Furthermore, the samples were classified into four subtypes of medulloblastoma, namely SHH, WNT, Group 3, and Group 4 (Supplementary Figure S2B). Then, we explored the expression of the five HOX-related signature genes. The results showed three HOXC genes (*HOXC4, HOXC5*, and *HOXC6*) mainly expressed in neurons and neuroepithelial cells. Moreover, these three HOXC genes are specifically expressed in WNT and Group 3 subtypes of medulloblastoma (Supplementary Figure S3C). To further investigate the protein expression of the hub genes, we analyzed immunohistochemistry (IHC) images from the Human Protein Atlas (HPA) database. HOXA6 and HOXC5 IHC staining was weak in the normal brain tissues, while glioma tissue exhibited strong HOXA6 and HOXC5 IHC staining (Supplementary Figure S3D). The results from HPA were consistent with PGs cohort. Additionally, the mIF in HPA revealed that HOXC4, HOXC5, and HOXC6 proteins were primarily expressed in nucleoplasm in the glioma cell line U-251MG (Supplementary Figure S3E).

## 4 Discussion

HOX family genes can function as both oncogenes and tumor suppressors. Still, they are generally pro-oncogenic in a more supportive role, both at the cellular and tumor levels, by driving cell proliferation, preventing apoptosis, and promoting angiogenesis, metastasis, and treatment resistance (Contarelli et al., 2020). Increased expression of HOX proteins has also been associated with poor prognosis of patients with gliomas, lung, liver, colorectal, head and neck, and ovarian cancers (Luo et al., 2019). Abnormal expression of certain members of the HOX family has been linked to cell proliferation and prognosis in gliomas (Tabuse et al., 2011). In recent decades, the survival rate of glioma patients has increased partly with the development of targeted and immunotherapy (Yang et al., 2022; Xu et al., 2020). While PGs still need accurate biomarkers for early diagnosis and a more precise prognosis. The HFGs’ role in pediatric gliomas (PGs) remains unclear. Therefore, a comprehensive and thorough investigation into the role of HOX family genes in PGs development is crucial. In this study, we aimed to explore the expression profile of the HFGs and their association with prognosis and potential clinical application in PGs.

By employing unsupervised consensus clustering and analyzing the transcriptome data of 39 HFGs, we identified two distinct subtypes characterized by differential HFG expression patterns. These subtypes exhibit associations with different prognoses, clinicopathological factors, genetic alterations, biological pathways, and TME characteristics. Notably, this is the first study to report such findings in the context of PGs. To identify hub genes within PGs, we utilized the RF algorithm and the nearest shrunken centroid algorithm. Through this process, we successfully identified a set of hub genes that are particularly relevant to PGs. Subsequently, based on these hub genes, we developed a HOX-related gene signature that is closely associated with the prognosis of PGs patients. To validate the prognostic value of this signature, we conducted analyses in both an internal testing set consisting of 191 patients and an independent cohort comprising 231 patients.

Fang L. et al. demonstrated that overexpression of *HOXB9* correlated with lymph node metastasis and poor survival in gliomas (Fang et al., 2014). Additionally, numerous studies have reported a positive correlation between the overexpression of HFGs and prognosis in various cancer types (Cantile et al., 2012; Bhatlekar et al., 2014b; Hur et al., 2014; Chiba et al., 2017). The consensus classification system identified ideal distinguishment in predicting OS in PGs. Specifically, patients belonging to the HOX-SI subtype exhibited a more favorable prognosis, while those classified as HOX-SII had worse clinical outcomes. Our findings are consistent with the majority of results in the literature, which suggests that overexpression of HFGs predicted a poor prognosis. In contrast, decreased expression of HFGs is indicative of a favorable prognosis in PGs.

In this study, we observed that the HOX-SII subtype had higher proportions of ependymoma, medulloblastoma, and HGG, which were associated with shorter OS. Ependymomas are the second most common type of malignant pediatric brain tumor. Forty percent of cases remain incurable, and the 5-year survival in infants with ependymomas is only 40%–52% (Gajjar et al., 2014; Sabnis et al., 2021). Medulloblastoma is the highest degree of intracranial malignancy of the glioma. In a clinical study of medulloblastoma in children, the 5-year event-free survival was 55.6%–70.2%, and overall survival was 66%–80% (Leary et al., 2021). For the WHO grade, our results show that HGG PGs cases were more likely than LGG PGs cases to be classified into HOX-SII subtype. Our findings indicated that HGG PGs cases were more likely to be classified into the HOX-SII subtype compared to LGG PGs. These results suggest that the HOX-SII subtype may be associated with more aggressive tumor characteristics and poorer outcomes in terms of survival.

A higher mutation burden in cancer has been associated with an increased abundance of neoantigens, which can elicit immune responses and potentially lead to favorable responses to immunotherapy in types of cancer (Tran et al., 2017; Roelands et al., 2020; Huang et al., 2021). Our results showed that *MUC4*, *AHNAK2*, *AHNAK*, *FLG2*, *MUC5AC*, *MUC3A*, and *MUC17* were the most common alterations in PGs. Additionally, we found that the HOX-SI subtype had a higher frequency of *BRAF* mutations, while TP53 mutations were more prevalent in the HOX-SII subtype. Interestingly, we also observed that the HOX-SII subtype had a lower mutation burden compared to the HOX-SI subtype. Our findings could imply that PGs patients with higher mutation burdens might have stronger immune infiltration, abundant immune checkpoint expression, a better prognosis, and strong antitumor responses to neoantigens.

In the tumor immune microenvironment (TME), the nontumor cells of stromal and immune cells dilute tumor purity, and a higher infiltration of immune cells is always associated with low tumor purity (Yoshihara et al., 2013). In our study, we observed that HOX-SI PGs showed lower tumor purity, higher immune activity, and more favorable clinical outcomes compared to the subtype of HOX-SII. Regarding the composition of the TME, our findings revealed that T cells, macrophages, and mast cells were the most common immune infiltrates in PGs. HOX-SI subtype had a higher abundance of most types of tumor-infiltrating immune cells than HOX-SII, including CD4 memory resting T cells, monocytes, M2 macrophages, resting mast cells, and neutrophils. It is worth noting that the M0 type of macrophage showed a higher composition in HOX-SII compared to HOX-SI in PGs tumors. Traditionally, naïve macrophages (M0) are functionally polarized into two subsets: M1 and M2 macrophages (Locati et al., 2020). However, Tang L et al. found M0 macrophages could also be polarized to regulatory macrophages (Mregs), which possess immunosuppressive function (Tan et al., 2022). Hence, the higher composition of M0 macrophages in HOX-SII may affect the response to immunotherapy, which needs to be further verified with experiments.

Expression levels of ICs can serve as predictors of response to ICIs therapy (Darvin et al., 2018). For the immune checkpoint molecular analysis, PDL1, PD1, and CTLA4 were higher expressions in HOX-SI, suggesting that patients with HOX-SI may have a more favorable response to anti-PD1/PDL1 or anti-CTLA4 therapies. On the other hand, HOX-SII tumors exhibited lower levels of ICs, indicating that they may be less likely to respond to IC inhibitors. This finding increases the difficulty of ongoing immunotherapy research, especially clinical studies focusing on immune targets for PGs that are refractory since more ependymoma, medulloblastoma, and HGG, which are in urgent need of promising immunotherapies (Jones et al., 2017; Mackay et al., 2018) were classified as HOX-SII. Further investigation is required to address the complexities of immunotherapy in PGs and identify alternative treatment strategies for HOX-SII subtypes.

To find out which genes play a key role in the HOX-related subtype classification, we screened the hub genes in 39 HFGs phenotype-based DEGs by RF and the nearest shrunken centroid algorithm principles. These hub genes were selected to construct a clinically applicable predictor for the HOX-related subtypes, and their performance was evaluated using AUC in the training and test sets. The five genes identified as key players were *HOXA6*, *HOXC4*, *HOXC5*, *HOXC6*, and *HOXA-AS3*. Previous studies have reported aberrant and overexpression of these genes in various cancer types, with implications for angiogenesis, metastasis, and treatment resistance. In colorectal cancer cells, upregulation of *HOXA6* promoted cell proliferation, migration, and invasion and inhibited apoptosis. *HOXA6* regulated apoptosis through the Bcl-2 signaling pathway and regulated migration and invasion through the EMT process (Wu et al., 2018). *In vitro*, *HOXA6* promoted cell proliferation, migration, and invasion in lung adenocarcinoma (LUAD) (Zhang et al., 2018). One study found that the suppression of *HOXA6* expression could reduce invasion tendency in glioma cell lines of U-118 and U-138 (Guo et al., 2016). The previous research identified *HOXC4* was strongly overexpressed in pediatric brain tumors, including ependymoma (Mendrzyk et al., 2006), medulloblastomas, glioblastoma multiforme, and juvenile pilocytic astrocytomas (Chakravadhanula et al., 2014). *HOXC5*, specifically enriched in tumor cells, has been significantly associated with poor prognosis in clear cell renal cell carcinoma (ccRCC) (Long et al., 2022). *HOXC5* could block angiogenesis and regulate pro-angiogenic/anti-angiogenic genes. *HOXC5* is expressed in quiescent endothelial cells (EC); its expression is diminished or absent in active angiogenic EC found in association with breast tumors (Rhoads et al., 2005). Many long noncoding RNAs (lncRNAs) have been identified as important cancer regulators. *HOXA-AS3*, an important long noncoding RNA (lncRNA), was found to be activated in lung adenocarcinoma (LAD) and supported cancer cell progression. Its expression was significantly higher in LAD tissues and A549 cells, and the knockdown of *HOXA-AS3* inhibited cell proliferation, migration, and invasion. Furthermore, *HOXA-AS3* increased the stability of *HOXA6* mRNA through the formation of an RNA duplex (Zhang et al., 2018). HOXC6, frequently overexpressed in multiple cancers, including glioma, was associated with poor prognosis in glioblastoma patients. Overexpression of *HOXC6* in glioma tissues and cell lines was linked to proliferation, clinical progression, and immune infiltrations. *HOXC6* might be a key factor in promoting tumorigenesis and glioma progression by regulating the EMT signaling pathway and might represent a novel immune therapeutic target in gliomas (Yu et al., 2021; Arunachalam et al., 2022; Huang et al., 2022). These findings underscore the significance of these genes in cancer development and suggest their potential as therapeutic targets or prognostic indicators in PGs.

Our analysis revealed that the patients classified into the HOX-SII subtype had a higher rate of medulloblastomas. Medulloblastomas comprise a biologically heterogeneous group of embryonal tumors of the cerebellum, which can be subdivided into four molecular subgroups: WNT, SHH, Group 3, and Group 4. Each subgroup has a distinct prognosis, biological behavior, and implications for targeted therapies (Northcott et al., 2019). Based on the single-cell data of 13 medulloblastomas, we observed that three HOXC genes (*HOXC4*, *HOXC5*, and *HOXC6*) were expressed in neurons and neuroepithelial cells, specifically within the WNT and Group 3 subtypes of medulloblastoma. This finding highlights the potential involvement of these HOXC genes in the pathogenesis and molecular characteristics of these particular medulloblastoma subtypes. Further research is warranted to explore the functional significance of HOXC genes in medulloblastoma development and their potential as therapeutic targets in specific subgroups.

To standardize the process of discriminating the subtypes of PGs, we developed and validated predictive formulas based on the expression levels of the five hub HOX-related genes. With expression sequencing data on these five genes, researchers could easily classify a PG into one subtype using the formulas. This classification enables the prediction of important clinical information, such as OS, TME characters, and immunotherapy responsiveness.

In the field of oncology, there has been a growing interest in developing classifiers that utilize various omics data to improve tumor classification and prognosis prediction. Bioinformatic analyses have played a crucial role in exploring and harnessing the wealth of information available in different omics datasets (Xie et al., 2022b; Wu et al., 2022; Zhang et al., 2022). These classifiers aid in better understanding tumor biology and guide personalized treatment strategies. By leveraging bioinformatic approaches and utilizing the expression profiles of the five hub HOX-related genes, our study contributes to the growing body of research focused on developing comprehensive classifiers for tumor subtyping and prognosis prediction. These findings enhance our understanding of PGs and offer potential avenues for targeted therapies and precision medicine in the future.

As for other diagnostic classification systems, studies mainly focused on adult gliomas. Wang et al. constructed seven stemness-related genes risk model to explore the immunotherapy response by multiple machine learning algorithms in adult gliomas (Wang Z. et al., 2021). Cluceru J et al. trained a classifier to evaluate the effects of training strategy and incorporation of biologically relevant images on predicting genetic subtypes with deep learning in diffuse gliomas (Cluceru et al., 2022). Due to the paucity of available published data on PGs, few diagnostic models for PGs have been reported until now. In our study, multiple datasets of PGs were integrated to explore the relationship between HOX genes and PGs. Furthermore, we were the first to combine utilized RF, the nearest shrunken centroid algorithm, and euclidean distance to construct a novel diagnostic classifier for PGs. However, there are some limitations to our study. Since the number of PGs patients who received immunotherapy is limited, further research is needed to confirm the association between the classifier and immunotherapy based on an immunotherapy cohort. Additionally, while we have validated the predictive performance in the internal testing set and an independent cohort, more *in vivo* and *in vitro* experimental validation need to be supplemented.

## 5 In conclusion

This study classified PGs into two subtypes based on HFGs expression perspective: HFG-low-expression (HOX-SI) and HFG-high-expression (HOX-SII). HOX-SI subtype showed high HFGs expression, a favorable prognosis, higher immune infiltration, and better responsiveness to immunotherapy. And this subtype comprised more astrocytoma and LGGs. On the other hand, the HOX-SII subtype demonstrated low HFGs expression, a dismal clinical outcome, lower immune infiltration, and poorer response to immunotherapy. And this subtype constituted more ependymoma, medulloblastoma, and HGGs. Furthermore, an innovative and clinically applicable PGs HOX-related subtype classifier was developed. This classifier has the potential to guide future mechanistic research and serve as a valuable tool for selecting appropriate therapies based on the predicted response of patients.

## Data Availability

Publicly available datasets were analyzed in the study. The data of genome sequencing, RNA-sequencing, survival, and clinical information of CBTTC, ICGC, CPTAC (CHOP, Cell 2020), and GSE73038 were separately from https://cbttc.org/, https://dcc.icgc.org/, https://www.cbioportal.org/, and https://www.ncbi.nlm.nih.gov/geo, respectively.
