# Supplementary material for: Identification and validation of a novel HOX-related classifier signature for predicting prognosis and immune microenvironment in pediatric gliomas
Source: Front Cell Dev Biol. 2023 Jul 21;11:1203650. doi: 10.3389/fcell.2023.1203650 (PMC10401438; doi:10.3389/fcell.2023.1203650)
Supplement: Supplementary file 1 [file DataSheet1.docx]

Supplementary Material

## Supplementary Figures


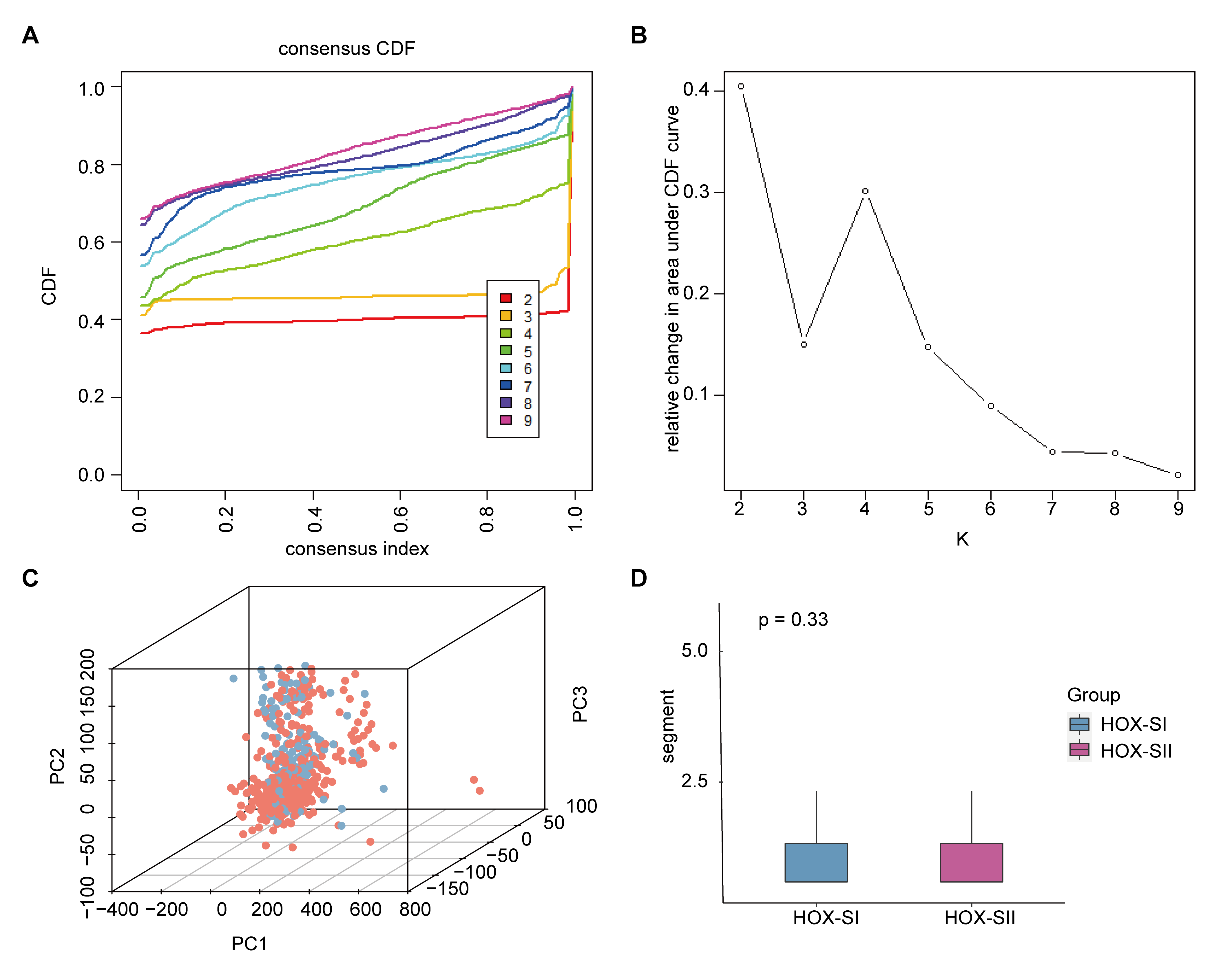


**Figure S1**. Cumulative distribution function curve (**A**) and delta area curve (**B**) based on HFGs expression profile in the PGs cohort. (**C**) Principal component analysis (PCA) shows a remarkable difference in transcriptomes between different HOX-related subtypes in the PGs cohort. The red points were HOX-SI, and the blue points were HOX-SII. (**D**) The comparison of CNV segment value between HOX-SI and HOX-SII. p value was inferred from Wilcoxon test. p<0.05 was considered statistically significant.


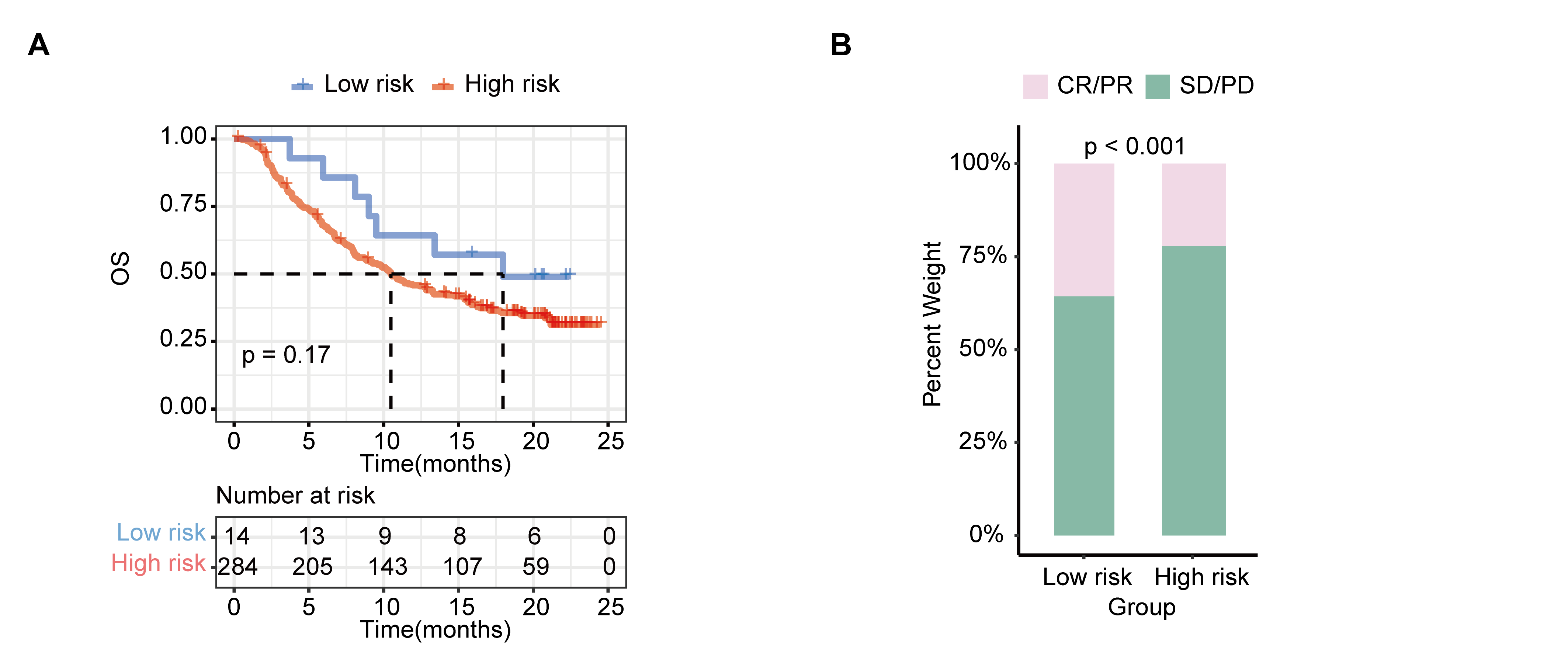


**Figure S2**. **Prognostic value of the HOX-related signature to immunotherapy.** (**A**) The Kaplan-Meir survival curve of OS between high- and low-risk groups in the IMvigor210 cohort. (**B**) Comparison of outcomes of immunotherapy between two groups in the IMgivor210 cohort. p<0.05 was considered statistically significant. OS, overall survival.


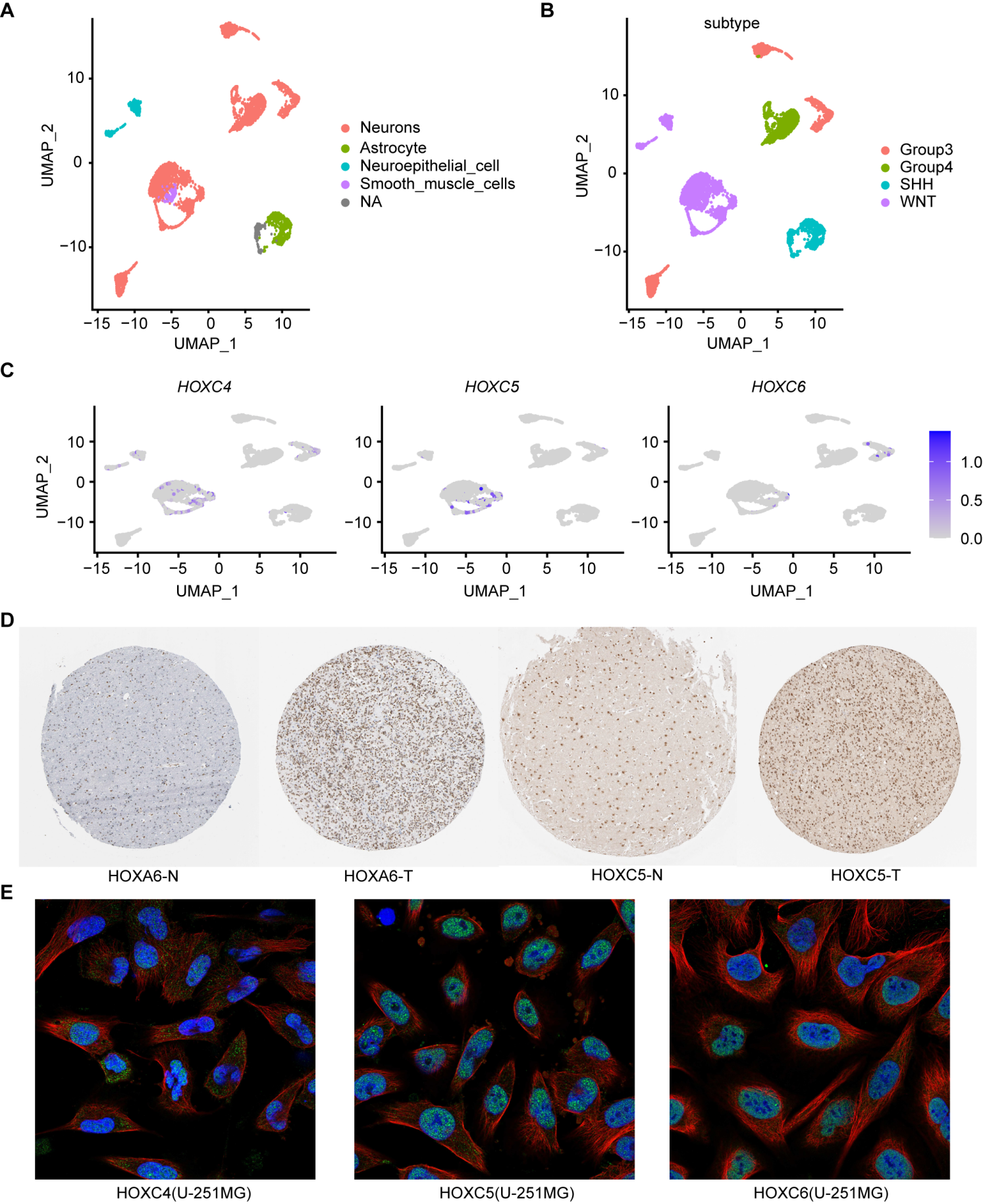


**Figure S3. Correlation between the HOX genes and Medulloblastoma subtypes. (A-C)** Cell annotations (**A**), Medulloblastoma subtype (**B**), and three HOXC genes (**C**) distributions in the single-cell transcriptome analysis. High HOXC cells were mainly located in the neurons, especially the subtypes of WNT and Group 3 cells. (**D**) Expression levels of HOXA6 and HOXC5 in cancerous and normal tissues. N, normal; T, tumor. (**E**) Expression of HOXC4, HOXC5, and HOXC6 in glioma cell line U-251MG. Green, Target protein; Blue, Nucleus; Red, Microtubules.
